# Supplementary material for: Prediction of Anthocyanin Color Stability against Iron Co-Pigmentation by Surface-Enhanced Raman Spectroscopy
Source: Foods. 2022 Oct 29;11(21):3436. doi: 10.3390/foods11213436 (PMC9658423; doi:10.3390/foods11213436)
Supplement: Supplementary file 1 [file foods-11-03436-s001.zip › foods-1967276-supplementary.pdf]

Supporting Information

Prediction of anthocyanin color stability against iron co-pigmentation by surface-enhanced Raman spectroscopy

Haochen Dai, Adam Forbes, Xin Guo, Lili He

Corresponding author: Lili He

Department of Food Science, University of Massachusetts Amherst, Amherst, MA 01003

lilihe@umass.edu

1. Silver Nanoparticle Characterization.

The calculated concentration of silver nanoparticles was 32.5 mg/L for this protocol based on UV-vis absorbance against a reference sample (commercial 40 nm silver nanospheres, 20mg/L, Nanocomposix, San Diego, CA, USA). The UV-vis spectrum of the synthesized nanoparticles displayed the characteristic surface plasmon resonance (SPR) band for silver, which was centered at 410 nm. In addition, no peaks located around 335 and 560 nm were detected, indicating the absence of nanoparticle aggregation (Figure S1).

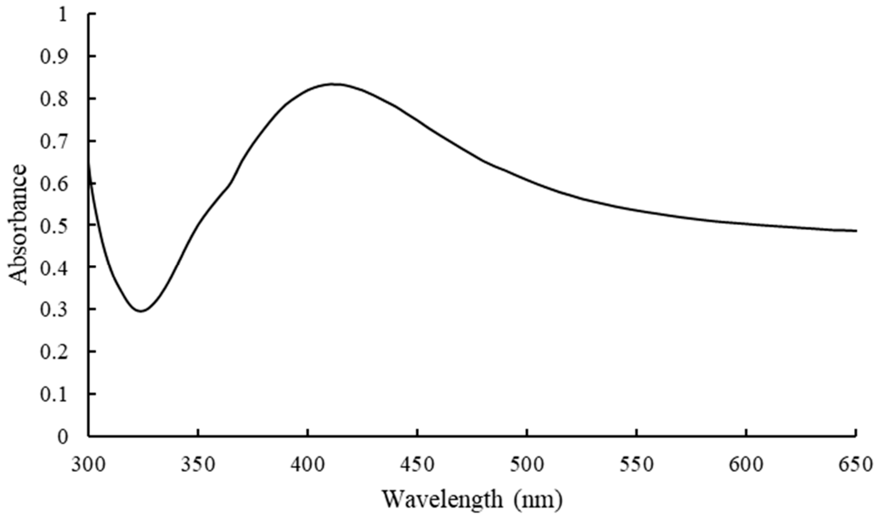

Figure S1. UV absorption spectra of silver nanoparticles fabricated in this study.

Dynamic light scattering (DLS) result was presented below in Table S1. The DLS was measured using a ZEN 3600 dynamic light scattering analyzer (Malvern Instrument Limited, Worcestershire, UK). The average particle size of silver nanoparticles was 67.66 ± 4.27 nm.

Table S1. Average diameter of silver nanoparticles by dynamic light scattering (DLS) analysis.

	Z-Ave (d.nm)
Measurement 1	64.56
Measurement 2	72.53
Measurement 3	65.89
Mean ± SD	67.66 ± 4.27

Scanning electron microscope (SEM) was conducted using an FEI Magellan 400 XHR-SEM (FEI, Hillsboro, US), and the images confirmed the shape of the silver nanoparticles to be spherical (Figure S2). Image J software (Java 1.8.0_345, National Institutes of Health, USA) was used to conduct size dimension and size distribution analysis for four individual SEM images. A total of 231 particles were analyzed. The result indicated that the average particle size was 54.5 ± 18.2 nm. Size distribution can be seen in Figure S3. 80% of the nanospheres fell in the diameter range of 30-70nm.

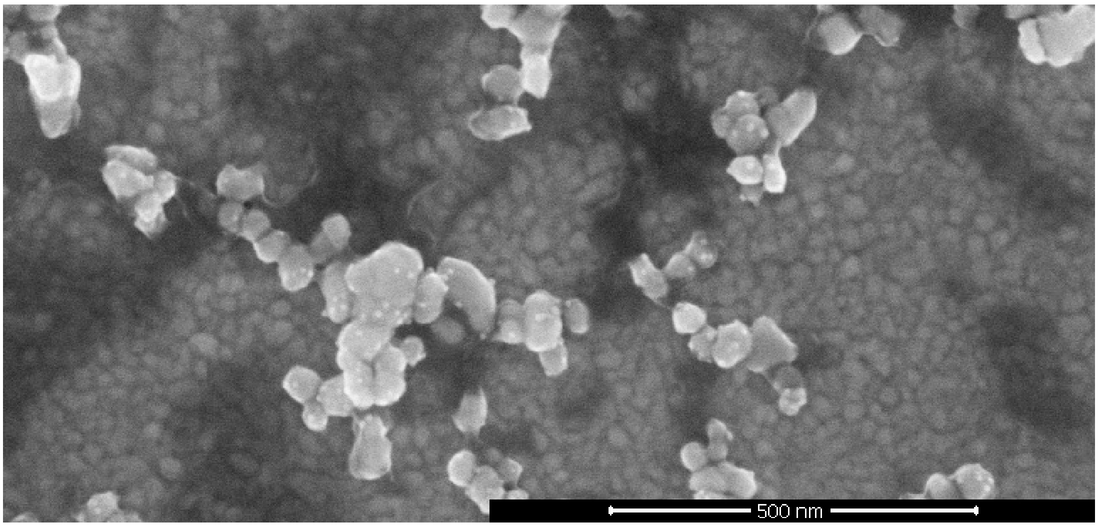

Figure S2. SEM image of silver nanoparticles (bright white).

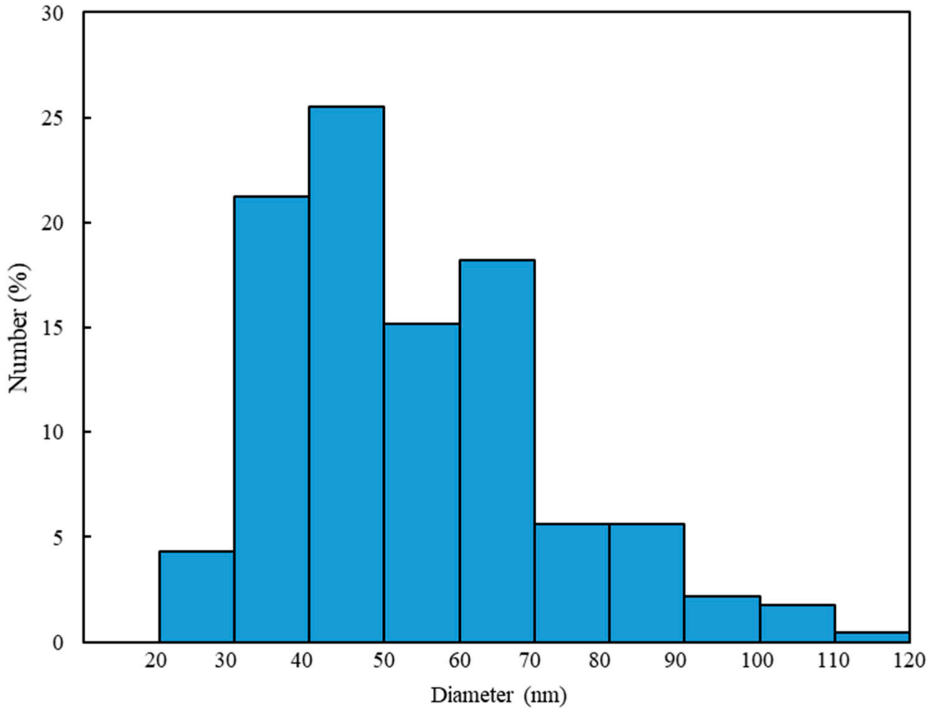

Figure S3. Nanoparticle size distribution analysis based on SEM images.

Furthermore, we tested the capability of the silver nanoparticles (CIST05) used in this study against commercial 40nm silver nanospheres (NanoComposix Inc., San Diego, CA, USA) and one of our homemade nanospheres (thermal reduced using sodium citrate) [1]. As can be seen in Figure S3, the silver nanoparticle from this study (CIST05) showed fewer background signals compared to the other two candidates. In the meantime, it generated more anthocyanin characteristic peaks when incubated with purple carrot extracts (Figure S4). The present data demonstrated the great SERS performance of using CIST05 silver for anthocyanin characterization.

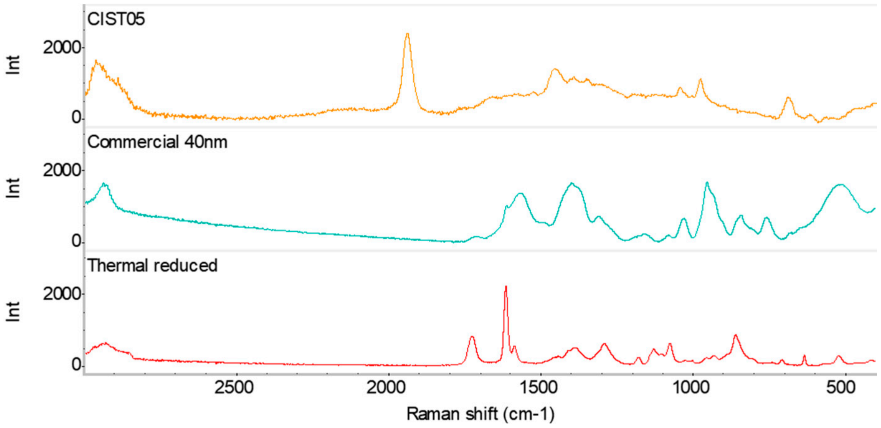

Figure S4. Stacked spectra of each blank silver nanoparticles (Averaged, Common scale).

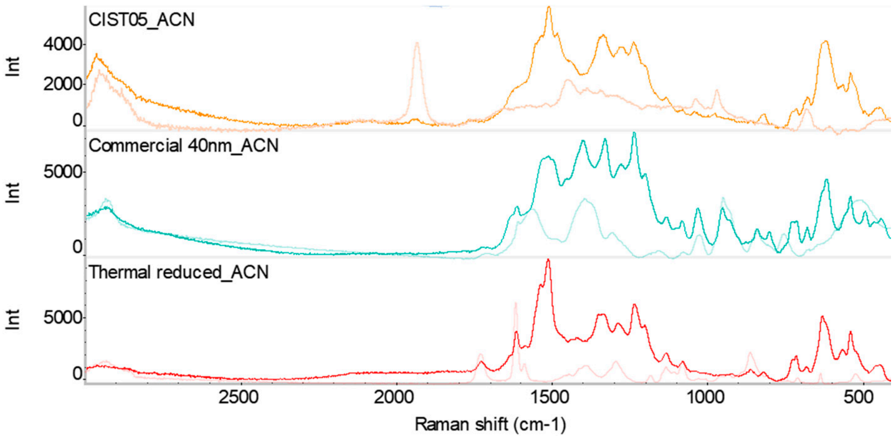

Figure S5. Stacked spectra of each silver nanoparticle incubated with purple carrot extract. (Averaged, Full scale, light-colored spectra represent their silver background signal).

2. PCA and PLSR supporting information.

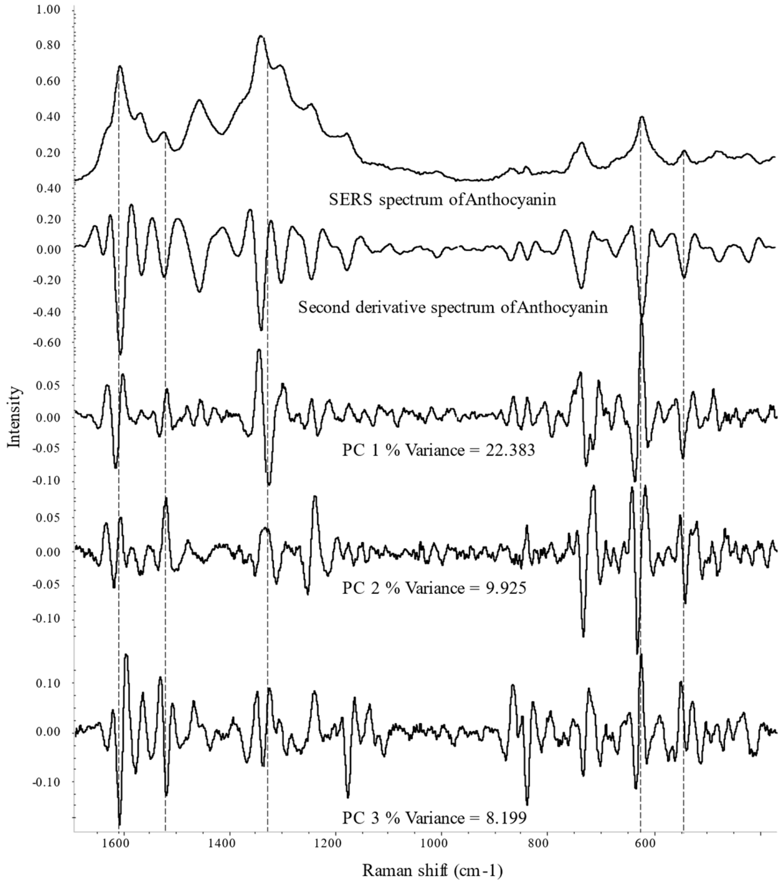

Figure S6. Principal component spectra of anthocyanin extracts.

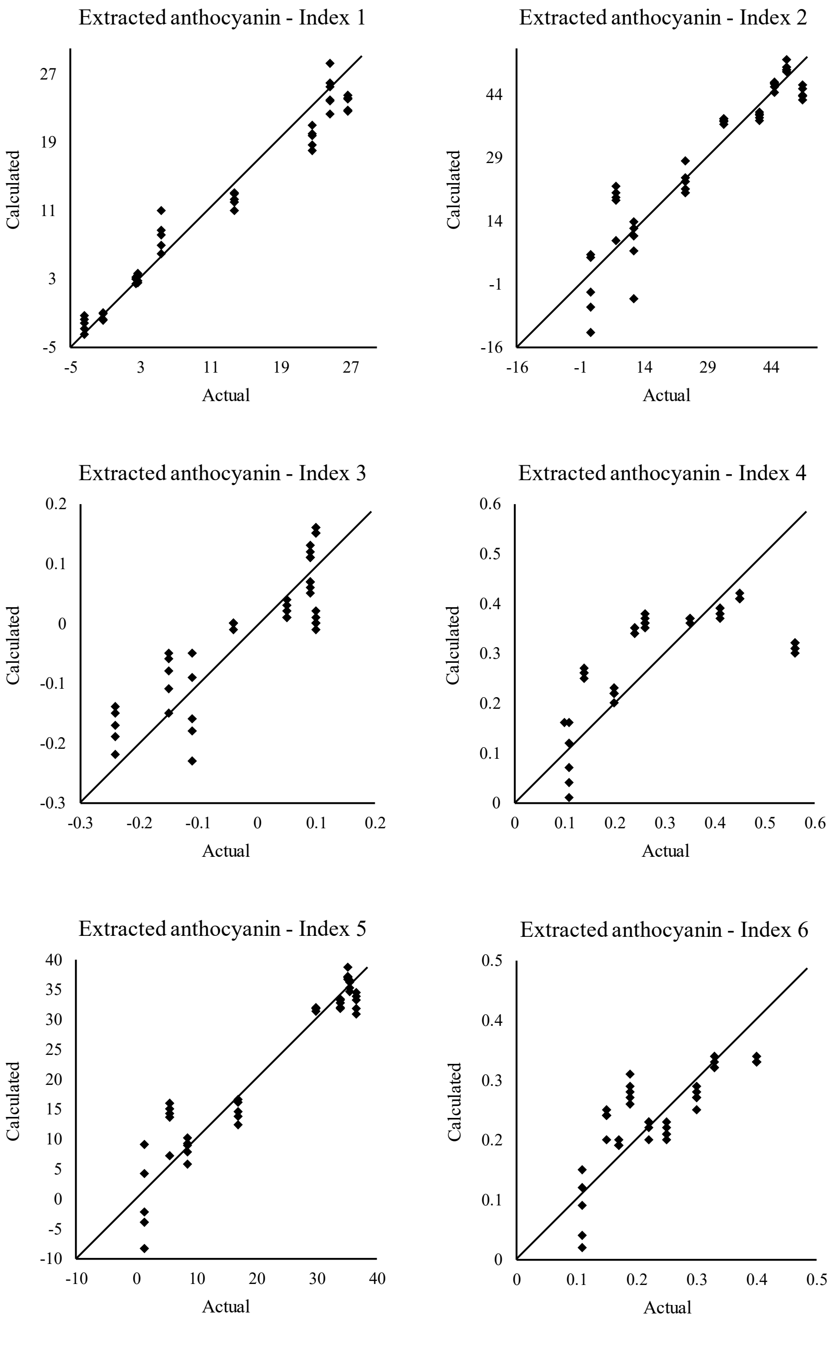

Figure S7. Actual vs. prediction PLS plot in external validation for each stabilization index.

Reference

1. Šileikaitė, A., Puišo, J., Prosyčevas, I., & Tamulevičius, S. (2009). Investigation of silver nanoparticles formation kinetics during reduction of silver nitrate with sodium citrate. Materials Science (Medžiagotyra), 15(1), 21-27.
